# Evaluation of rKDDR-Plus ELISA in Dogs with Different Clinical and Serological Profiles of *Leishmania infantum* Infection

**DOI:** 10.3390/ani16193097

**Published:** 2026-10-02

**Authors:** Clara Jiménez-Fortunato, Ana Carolina Amado-Gomes, Marta Baxarias, Andrea Murillo-Picco, Lilian Lacerda Bueno, Ricardo Toshio Fujiwara, Laia Solano-Gallego

**Affiliations:** 1 Departament de Medicina i Cirurgia Animals, Facultat de Veterinària, Universitat Autònoma de Barcelona (UAB), 08193 Bellaterra, Spain; jimenezfclara@gmail.com (C.J.-F.); martabaxcan@gmail.com (M.B.); dianaandrea.murillop@gmail.com (A.M.-P.); 2 Departamento de Parasitologia, Instituto de Ciências Biológicas, Universidade Federal de Minas Gerais (UFMG), Belo Horizonte 31270-901, Brazil; carolamadobiologa@hotmail.com (A.C.A.-G.); llbueno@icb.ufmg.br (L.L.B.); fujiwara@icb.ufmg.br (R.T.F.)

**Keywords:** canine leishmaniosis, recombinant protein, serodiagnosis

## Abstract

Most *Leishmania infantum* infected dogs remain healthy, with low parasitic loads and negative to low antibody levels, making detection challenging. rKDDR-plus has shown outstanding diagnostic performance in ELISA but has never been tested in dogs thoroughly characterized by physical examination and routine laboratory tests. This study aimed to evaluate the diagnostic performance of rKDDR-plus ELISA in dogs with different *L. infantum* infection states (*n* = 94), classified as healthy seronegative (*n* = 25), healthy seropositive (*n* = 54), and sick (*n* = 15). In total, 37 seropositive dogs were classified as low and 32 as high. Diagnostic performance was evaluated in five groups: (G1) all dogs, (G2) healthy seronegative + sick (*n* = 40), (G3) healthy seronegative + healthy seropositive (*n* = 79), (G4) healthy seronegative + high seropositive (*n* = 57), and (G5) healthy seronegative + low seropositive (*n* = 62). Negative percent agreement was 100% in all groups, while positive percent agreement was perfect in G2 (100%), followed by G4 (96.88%), G1 (82.61%), G3 (77.78%), and G5 (70.27%). Accuracy ranged from 85.14% to 100%, and the area under the ROC curve (AUC-ROC) was as follows: G2 (1.000), G4 (0.978), G1 (0.951), G3 (0.937), and G5 (0.928). Agreement (kappa) was almost perfect in G2 and G4, and substantial in the remaining groups. In conclusion, rKDDR-plus ELISA shows overall good diagnostic performance.

## Simple Summary

*Leishmania infantum* infection in dogs is complex, as different clinical and immunological profiles coexist in an endemic area. For instance, infected dogs may show no clinical signs and appear healthy, while others develop disease. In fact, clinically healthy infected dogs are the most challenging to detect. However, it is important to detect and control these dogs, as they can maintain and spread the infection and may develop disease later on. rKDDR-plus is a recombinant protein designed to improve the performance of serological tests. Nevertheless, it has not been thoroughly evaluated in dogs classified not only by physical examination but also by routine blood and urinary laboratory tests. The objective of this study was to assess the diagnostic performance of the rKDDR-plus-based antibody detection test in dogs with different *L. infantum* infection states. The results showed that the test reliably identified antibody levels in high seropositive dogs while also yielding negative results in healthy, antibody-negative dogs. However, its ability to detect antibodies was reduced in dogs with low antibody levels. Overall, rKDDR-plus represents a useful diagnostic tool that could be applied not only in research and epidemiological studies, but also in routine veterinary practice.

## 1. Introduction

Canine leishmaniosis is an important zoonosis caused by the obligate intracellular protozoan *Leishmania infantum* [1,2]. Most sick dogs have mild to moderate disease, although some also present with severe to very severe disease that is characterized by renal alterations [3]. The LeishVet group classified sick dogs into four stages to better assess their treatment, monitoring, and prognosis [4]. Interestingly, all dogs with moderate to very severe disease develop antibodies against *L. infantum* antigen [4] and are the ones that maintain and spread the infection [5]. However, most infected dogs do not develop the disease, remaining as healthy infected dogs [6,7]. These dogs are challenging to detect; in addition to presenting no clinical signs or laboratory abnormalities, they usually have a low parasitic load and low or even negative antibody levels [6,8]. This means that they can be misclassified depending on the sample used [9,10], the sequence amplified by PCR [9], and the serology test used [10].

To successfully diagnose the infection, visualization of the amastigotes by cytology or histology, in combination with immunohistochemistry, serology, and molecular techniques such as PCR, is commonly used [11,12]. However, all these diagnostic techniques present limitations and disadvantages, as previously reviewed [13]. Observation of amastigotes has variable sensitivity [14] and requires trained and expert personnel [15,16]. Molecular techniques not only require sophisticatedly equipped laboratories [13,16] and well-trained staff [16], but they are also expensive [13]. Although they can have high sensitivity, it differs depending on the sample analyzed and the method used [13].

On the other hand, the rapid immunochromatographic test is a qualitative serological method that does not need highly equipped laboratories [16] and trained personnel but has variable diagnostic performance compared with quantitative serology [13,16]. Therefore, a negative result in a clinically suspect dog and a positive result in any dog should be confirmed by a quantitative serological method [1]. Regarding quantitative serology, although the enzyme-linked immunosorbent assay (ELISA) appears to have higher sensitivity for detecting healthy seropositive dogs than the immunofluorescence antibody test (IFAT) [13], the latter is classically considered the reference serological method due to its sensitivity and specificity near 100% [1,13].

Serological methods may employ crude promastigote antigens, soluble parasite extracts, or recombinant proteins, each exhibiting distinct performance characteristics and inherent limitations [13]. Nevertheless, additional antigens continue to be investigated and developed in the ongoing search for optimal diagnostic performance. For instance, from 2016 to 2021, the diagnostic performance of 50 antigens was tested in ELISA [17]. Considering that dogs with mild disease and healthy seropositive dogs are challenging to detect [13], there is a need for improving serological tests, and the development of recombinant proteins could fill this need. These proteins are designed to increase the diagnostic test’s specificity [18] and sensitivity [19].

rKDDR-plus is a recombinant protein derived from *L. infantum* kinesin [20,21]. Kinesins are a family of motor proteins involved in mitosis [22,23], intracellular transport [23], cell morphology, and motility [22]. rKDDR-plus is formed by a tandem repeat of 39 amino acids repeated 15.3 times, making a protein of 1788 amino acids [20]. Tandem repeat proteins potentially contain highly antigenic B-cell epitopes in protozoa [24].

rKDDR-plus has been tested with samples from human visceral leishmaniasis patients [20], and *L. infantum*-infected cats [25], and dogs [20,21,26,27,28] with good sensitivity and specificity. However, dogs were classified as symptomatic or asymptomatic only based on physical examination [20,21,26,27,28]. Moreover, it has never been tested on samples from dogs characterized by a thorough physical examination, blood and urinary routine laboratory tests, and degree of antibody levels. Therefore, this study aimed to explore the diagnostic performance of the rKDDR-plus serological ELISA test in dogs in different states of *L. infantum* infection classified according to their clinical and immunological status based on a physical examination, blood and urinary routine laboratory tests, and antibody levels. This study evaluates concordance with the reference serological method (*L. infantum* antigen-coated ELISA). The results from the present study indicate that the rKDDR-plus ELISA is a reliable diagnostic tool, particularly for detecting antibodies in high seropositive dogs and providing negative results in seronegative animals, although it has some limitations in detecting healthy seropositive and low seropositive dogs.

## 2. Materials and Methods

### 2.1. Dogs

Residual serum samples from 94 dogs from different areas of Spain were collected between 2020 and May 2022 by veterinarians from primary-care veterinary clinics and, occasionally, animal shelters, as part of previously published prospective clinical studies [6,29,30]. Dogs of either sex (intact or neutered), any breed (purebred or mixed-breed), aged >1 year, and with available clinical and laboratory data were eligible for inclusion. Dogs were classified as healthy seronegative (*n* = 25), healthy seropositive (*n* = 54), and sick (*n* = 15) based on the results of thorough physical examination, blood and urinary routine laboratory tests (complete blood count, serum biochemistry, and urinalysis with urinary protein creatinine ratio), and measurement of antibody levels by an *in-house* ELISA with *L. infantum* antigen-coated plates, as described below [6]. Dogs were included as sick when they presented clinical signs and/or clinicopathological abnormalities compatible with leishmaniosis, along with a low to high antibody level. These dogs were further staged according to the LeishVet staging system [4]. Dogs without clinical signs and clinicopathological abnormalities but with detectable anti-*L. infantum* antibodies were included as healthy seropositive [6]. Dogs without clinical signs, clinicopathological abnormalities, or detectable anti-*L. infantum* antibodies were included as healthy seronegative. Exclusion criteria for all dogs included no prior diagnosis of clinical leishmaniosis, vaccination against canine leishmaniosis, and no anti-*Leishmania* drug administration [6,29,30]. As samples were selected from previous studies and not through population-based sampling, the study population was not intended to represent the prevalence of *L. infantum* seropositivity in the Spanish canine population. Moreover, 37 out of the 69 positive sera were classified as low seropositive (<150 EU), while 32 were considered high (≥150 EU). Additionally, dogs were classified as young (<2 years), adults (between 2 and less than 8 years), and geriatric (more than 8 years).

### 2.2. Leishmania infantum Antigen-Coated ELISA

The antibody level was measured by an in-house *L. infantum* antigen-coated ELISA (diagnostic ELISA), as previously mentioned [6]. First, plates were coated with a final concentration of 20 µg/mL of sonicated *L. infantum* promastigotes. Then, samples were added at a 1:800 dilution in phosphate-buffered saline (PBS)-0.05% tween 20 and 1% skimmed milk. After 1 h of incubation at 37 °C, the plates were washed 3 times with PBS-0.05% tween 20 for 3 min each and for 1 min with PBS. Later, peroxidase-conjugated protein A at a dilution of 1:30,000 (Peroxidase Conjugate Protein A; Merck KGaA, Darmstadt, Germany) was added to the wells and incubated for 1 h at 37 °C. Next, washes were repeated, and the reaction was developed using o-phenylenediamine dihydrochloride (OPD) (SIGMAFAST OPD; Merck KGaA, Darmstadt, Germany). Finally, the reaction was stopped using 2.5 M H_2_SO_4_, and the plates were read using an ELISA reader (MB-580 HEALES; Shenzhen Huisong Technology Development Co., Ltd., Shenzhen, China) at 492 nm.

Positive and negative controls and a blank were added to each plate, and all samples were run in duplicates. Positive controls included a strongly seropositive serum sample and a low-positive calibrator serum sample. Results were expressed as ELISA Units (EU) concerning a calibrator, a positive sample arbitrarily set to 100 EU. The cut-off was established at 35 EU [31]. Moreover, positive sera were classified as low when the positivity percentage was lower than 150 EU and high when it was equal to or higher than 150 EU [32]. A two-fold dilution of the high seropositive samples was performed, starting with the 1:800 dilution and continuing for between seven and 11 dilutions (endpoint ELISA). Results were expressed relative to a calibrator arbitrarily set at 100 EU with an optical density (OD) value of 1 at 1:800 dilution. The three mean values of OD of each sample close to the calibrator’s OD were chosen to calculate the EU result using the formula (sample OD/calibrator OD) × 100 × dilution factor [6].

### 2.3. rKDDR-Plus ELISA

The rKDDR-plus ELISA was performed as previously described [20,21] with some modifications. First, plates (Costar, Cambridge, MA, USA) were coated with a final concentration of 100 ng/mL of rKDDR-plus in carbonate buffer and incubated overnight at 4 °C. Then, plates were washed five times with PBS-0.05% tween 20. After blocking the plates with 2% bovine serum albumin in PBS for 1 h at 37 °C, serum or plasma samples were added at a 1:100 dilution in PBS-0.05% tween 20 in duplicates and incubated overnight at 4 °C. Afterward, washes were repeated, and anti-dog IgG horseradish peroxidase (Sigma-Aldrich, Saint Louis, MO, USA) at a 1:2500 dilution was added to the wells. After one hour at room temperature, plates were washed again, and the reaction was developed using the substrate solution [Na_2_HPO_4_ 0.2 M, citric acid 0.1 M, o-phenylenediamine dihydrochloride (OPD) 0.05% and H_2_O_2_ 0.1%]. The reaction was stopped after 15 min in the dark with 2 M H_2_SO_4_. Finally, optical densities were measured at 492 nm using a spectrophotometer (VersaMax, Molecular Devices, San Jose, CA, USA).

Three negative controls, two positive controls, and three blanks were added to each plate in duplicates. The cut-off of each plate was calculated as the mean optical density of the negative controls plus two times the standard deviation of these samples. The reactivity index (RI) of each sample was calculated as the mean value of the optical density divided by the cut-off. When the RI was above 1.000, the samples were considered positive. Conversely, when the RI was below 1.000, the samples were classified as negative.

### 2.4. Statistical Analysis

The present study adhered to the veterinary STROBE statement recommendations [33]. A descriptive analysis of the groups was performed using the software Jamovi 2.3.18 (Sydney, Australia) [34], using the packages R Core Team (Version 4.1) [35], and Visualizing Categorical Data [36]. Fisher’s exact test or chi-square tests were performed to describe the qualitative variables sex (female/male), spayed (yes/no), breed (purebred/mixed breed), and age (young/adult/geriatric). The Shapiro–Wilk test was used to assess the normality of the quantitative data (age, diagnostic and endpoint ELISAs, and weight). Then, they were assessed by the Student *t*-test or the Mann–Whitney U-test if two groups were being compared. The Kruskal–Wallis H-test was calculated when more than two groups were compared, and afterward, the Dwass–Steel–Critchlow–Fligner test was used for pairwise comparison. A *p*-value below 0.05 was considered statistically significant.

The diagnostic performance was evaluated in five groups (G1–5): (G1) all dogs (*n* = 94), (G2) healthy seronegative + sick (*n* = 40), (G3) healthy seronegative + healthy seropositive (*n* = 79), (G4) healthy seronegative + high seropositive (*n* = 57), and (G5) healthy seronegative + low seropositive (*n* = 62).

Considering the results from the in-house *L. infantum* antigen-coated ELISA (diagnostic ELISA) as the reference serological method, the diagnostic parameters measured in each group were: (1) positive percent agreement (PPA), (2) negative percent agreement (NPA), (3) accuracy, (4) area under the curve—receiver operating characteristic (AUC—ROC), (5) Youden index, (6) kappa index, (7) predictive positive value (PPV), and (8) negative predictive value (NPV). As infection was not confirmed by PCR or visualization of amastigotes in lesions in all dogs, the terms PPA and NPA are more suitable in this case than sensitivity and specificity [37,38]. Accuracy [39], PPV, and NPV [37,38] were calculated considering the seroprevalence of 5%, 10%, 20%, 30%, and 50% observed in endemic areas of Spain [40]. A 95% confidence interval was calculated for PPA, NPA, accuracy, PPV, and NPV in each group of dogs using the statistical software package for biomedical research MedCalc, Version 23.2.1 [41]. The kappa index was used to determine the agreement level between tests, considering κ < 0 as poor, 0 ≤ κ ≤ 0.2 as slight, 0.21 ≤ κ ≤ 0.4 as fair, 0.41 ≤ κ ≤ 0.6 as moderate, 0.61 ≤ κ ≤ 0.8 as substantial, and 0.81 ≤ κ ≤ 1 as almost perfect [42].

Receiver operating characteristic (ROC) curve analyses were performed using the continuous relative index (RI) values obtained with the rKDDR-plus ELISA, with the serological classification obtained by the reference ELISA used as the state variable. ROC analyses were performed separately for each study group (G1–G5). The area under the ROC curve (AUC) was calculated together with its standard error (SE) and 95% confidence interval (CI) using the nonparametric procedure implemented in BM SPSS Statistics for Windows, Version 30.0. IBM Corp: Armonk, NY, USA. As AUC values are theoretically bounded between 0 and 1, asymptotic confidence limits falling outside this range were constrained to the corresponding theoretical boundary for reporting. The optimal RI cutoff for each ROC analysis was determined as the value maximizing the Youden index (J = PPA + NPA − 1), and the corresponding PPA and NPA were reported. The RI cutoff of 1.000 used to classify rKDDR-plus ELISA results as positive or negative was prespecified and was not derived from the ROC analyses. Therefore, the ROC-derived optimal cutoffs were considered exploratory. The same dataset was used to derive the ROC-optimal cutoffs and to estimate their performance. A significance level of *α* = 0.05 was considered.

## 3. Results

### 3.1. Clinical and Serological Data

Four out of 15 sick dogs were classified as LeishVet stage I and 11 as stage IIa. Additionally, seven dogs were classified as young, 54 as adult, and 21 as geriatric. Unfortunately, age data were missing for 12 dogs. Qualitative and quantitative clinical data of the dogs are summarized in Table 1.

The distribution of rKDDR-plus ELISA RI values according to the clinical and serological profiles of the dogs is shown in Figure 1. Healthy seronegative dogs showed RI values below the prespecified cutoff, whereas a broader distribution of RI values was observed in healthy seropositive and sick dogs, with most values above the cutoff.

There was no relation between antibody level (low/high) and clinical condition (healthy seropositive/sick) (Fisher’s exact test: *p* = 0.572). No association was identified either between LeishVet stage (I/IIa) and antibody level (Fisher’s exact test: *p* = 0.282). Moreover, no differences were observed between the median values of the EU endpoint of LeishVet stage I (112 EU (interquartile range, IQR: 99.1 EU)) and IIa (339 EU IQR: 773.6 EU) (Mann–Whitney U test: W = 10, *p* = 0.138).

No differences were observed in EU when dogs were separated according to sex, reproduction status, breed, and age. However, statistically significant differences in EU were shown when dogs were divided according to their clinical condition. The median of EU obtained from the endpoint ELISA of the healthy seronegative dogs [16.6 EU (10.6)] was significantly lower when compared to healthy seropositive dogs [176.1 EU (313.6)] (Kruskal–Wallis H-test: χ^2^ = 54.26, *p* < 0.001) and sick dogs [202.7 EU (327.2)] (*p* < 0.001). No differences were obtained between the healthy seropositive and sick dogs regarding EU (*p* = 1) (Table 2).

A higher proportion of mixed breeds (78.8%) compared to purebreds (54.2%) were classified as seropositive by rKDDR-plus ELISA (Chi-square: χ^2^ = 5.16, df = 1, *p* = 0.023). *L. infantum* antigen-coated ELISA also indicated a higher proportion of positive mixed breeds (87.9%) compared to purebreds (72.9%), but this difference was not statistically significant (chi-square: χ^2^ = 2.64, df = 1, *p* = 0.104). No other differences were found regarding the seroreactivity rate obtained by rKDDR-plus ELISA and *L. infantum* antigen-coated ELISA, and sex, reproductive status, and age (Table 2).

### 3.2. Diagnostic Performance of rKDDR-Plus ELISA

Diagnostic parameters calculated in each group to analyze rKDDR-plus ELISA performance are shown in Table 3. The PPA reached an optimum (100%) in G2, while this parameter decreased in the rest of the groups (G1 82.6%, G3 77.78%, G4 96.88%, G5 70.27%). The NPA was perfect in all groups (100%).

Youden index results were as follows: G2 (1), G4 (0.97), G1 (0.83), G3 (0.78), and G5 (0.70).

The level of agreement between *L. infantum* antigen-coated ELISA and rKDDR-plus ELISA was almost perfect in G2 (1) and G4 (0.965), while it was substantial in the rest of the groups (G1 0.716, G3 0.689, G5 0.656).

PPV, NPV, and accuracy were calculated using different seroprevalence scenarios of 5%, 10%, 20%, 30% and 50% (Table 4). PPV values do not change between different seroprevalences, as the rKDDR-plus ELISA did not exhibit any false positive results. The highest NPV was recorded when the healthy seronegative dogs were combined with the sick dogs (G2) (100%), and did not change between the different seroprevalences used. The lowest NPV was achieved in G5 at 50% seroprevalence (77.08%). Accuracy was perfect in G2 (100%) and decreased to 85.14% in G5 at 50% seroprevalence.

Analysis of the ROC curves and AUC (Figure 2) suggests the good diagnostic performance of rKDDR-plus in all groups, as all the AUC values indicated a high accuracy (Table 5).

## 4. Discussion

The present study supports the good diagnostic performance of the rKDDR-plus ELISA in canine samples, especially for high seropositive and healthy seronegative dogs. The almost perfect agreement observed among high seropositive and clinically sick dogs demonstrates that the rKDDR-plus ELISA can reliably differentiate high seropositive dogs from healthy seronegative dogs in concordance with the reference ELISA. Likewise, the AUC-ROC (0.978) values obtained in high seropositive dogs indicate high diagnostic accuracy [43], confirming the robustness of the assay. Although a PPA of 100% and an AUC of 1.0 were observed in sick dogs, these findings should be interpreted cautiously because they were based on a small number of affected animals (*n* = 15). The wide confidence intervals reflect the limited precision of these estimates and highlight the need for validation in larger cohorts of sick dogs with different clinical stages.

It is important to highlight that diagnostic performance decreases with respect to the detection of healthy seropositive and low seropositive dogs. Healthy seropositive dogs usually exhibit low antibody levels [6,31] and, therefore, are more difficult to detect. This reduced capacity to detect low antibody levels may be partly explained by the characteristics of the antigen used. In general, when whole antigen is employed in serological tests, there is higher sensitivity but lower specificity [13] compared with recombinant proteins such as rKDDR-plus. It is a recombinant protein derived from *L. infantum* kinesin, specifically designed to enhance specificity and minimize serological cross-reactivity [20]. Because rKDDR-plus contains only selected immunodominant epitopes rather than the complete antigenic repertoire of *L. infantum*, dogs producing antibodies against other *Leishmania* antigens, particularly those with low antibody levels, may not be detected [20].

In this study, PPV and NPV represent the probabilities that positive and negative rKDDR-plus ELISA results, respectively, agree with the classification obtained using the reference ELISA. These predictive values are more important for the daily routine of veterinary clinics and hospitals than sensitivity and specificity. While sensitivity and specificity are stable statistics of a test, NPV and PPV change with the prevalence of infection. Therefore, PPV rises and NPV falls with increasing prevalence, whereas the opposite occurs as prevalence decreases. Thus, these statistics change according to the geographical region [37,38]. In the present study, using several seroprevalence scenarios reported in Spain [40], the PPV of the rKDDR-plus ELISA was 100% across all groups, reflecting the absence of false-positive results and the excellent NPA (100%). This indicates that, under the epidemiological conditions evaluated in this study, a positive result is highly predictive of true antibody levels against *L. infantum*. Conversely, NPV ranged from 77.08 to 100%, depending on the clinical–serological profile and antibody level and seroprevalence scenario. The NPV was lower in dogs with low antibody levels or healthy seropositive dogs, in which antibody detection may be below the assay’s detection threshold. This pattern is consistent with the results reported by Siqueira et al., 2021, who obtained similar PPV (95.08%) and NPV (98.32%) values, but they calculated them directly from their sample rather than based on seroprevalence [20].

In the present study, the PPA (82.6%) and accuracy (91.3–99.1%) were lower than those reported in the most recent studies evaluating this recombinant protein, which described PPA values of 96.7% and 97.2% and accuracy values of 99.2% and 97.9%, respectively [20,21]. In the present work, dogs from different states of infection were included. They were characterized through physical examination and routine laboratory tests, including complete blood count, serum biochemistry, and urinalysis, providing a more accurate clinical assessment of the assay’s diagnostic performance and reflecting the clinical and serological diversity observed in routine veterinary practice. In contrast, in the previous studies described above, dogs were classified as asymptomatic or symptomatic based only on physical examination and positive results in serology, molecular and/or parasitological tests [20,21]. However, direct comparisons among studies should be interpreted with caution, as differences in study design and population characteristics may strongly affect diagnostic performance. Furthermore, Gondim et al. (2022) showed a lower sensitivity of the rKDDR-plus ELISA (74%), as their reference method was a positive result in PCR from bone marrow and conjunctiva or visualization of amastigotes in bone marrow [27]. These discordant results probably reflect differences in parasite burden, tissue distribution of the parasite, and the host immune response during different stages of infection. Nevertheless, some seropositive dogs may test PCR-negative [6,10], and conversely, some PCR-positive dogs can yield negative serological results [8,10].

Despite these variations, the present study achieved a NPA of 100%, in agreement with the specificity calculated in previous studies (95.52% and 97.5%) [20,21]. Taken together, these results confirm that the rKDDR-plus ELISA performs robustly as a confirmatory diagnostic tool in endemic areas, providing high reliability for positive results while maintaining a strong capacity to correctly identify seronegative dogs among clinically healthy animals. Therefore, the rKDDR-plus ELISA may represent a valuable serological diagnostic tool not only for research and epidemiological studies but also for routine use in veterinary diagnostic laboratories and clinical practice.

We would like to highlight that the samples used in this study have not been tested for other parasites for which serological cross-reactivity has been documented, such as *Babesia* sp. [44,45], *Ehrlichia* sp. [44,46], *Trypanosoma cruzi* and *Toxoplasma gondii*. However, the serological cross-reactivity with *Ehrlichia canis* or *Babesia* species is uncommon, since they are phylogenetically not closely related to *L. infantum* [47,48,49]. All dogs included in this study were from Spain, where, to date, no autochthonous cases of *Trypanosoma* infection in dogs have been documented, and the human transmission in this non-endemic area is non-vectorial [50]. Finally, the serological cross-reactivity with *Toxoplasma gondii* is not commonly documented in the literature [47,48]. Moreover, the specificity of the *L. infantum* antigen-coated ELISA used in this study has been validated [47]. Furthermore, regarding cross-reactivity with the rKDDR-plus ELISA, previous studies tested the performance of this test with positive samples of *Babesia* sp., *Ehrlichia* sp., and *T. cruzi*, and presented a low percentage of cross-reactivity [20,21]. Therefore, although this limitation should be acknowledged, its impact on the present findings is likely to be minimal.

Higher antibody levels and a greater proportion of highly seropositive dogs would be expected in sick dogs than in healthy seropositive dogs, as previously reported [6,51,52,53]. However, this pattern was not observed in the present study. This discrepancy may be attributable to the limited number of sick dogs included (only 15), and only dogs classified as LeishVet stages I and IIa were represented. It has been demonstrated that dogs with severe disease present higher antibody levels than dogs with mild to moderate disease [3,54]. Likewise, no significant differences in EU values or antibody levels (high/low) were detected between LeishVet stages I and IIa, in contrast to previous reports that described increasing antibody levels with increasing disease severity [55,56]. This finding may be explained by the small sample size, particularly the low number of dogs in stage I (*n* = 4). Inclusion of dogs from more advanced clinical stages might have resulted in more pronounced differences [6,55,57].

Another limitation of this study was that infection was not confirmed by parasitological or molecular methods in all cases, and it would be appropriate to have other reference diagnostic tests. Additionally, the negative control sera used to calculate the cut-off value for each rKDDR-plus ELISA plate originated from Brazil. As the epidemiological background can vary geographically, influencing the diagnostic cut-off [58,59], the use of control sera from the same endemic area as the tested samples, such as Spain or the Mediterranean basin, would likely provide a more accurate and representative cut-off.

## 5. Conclusions

Overall, the rKDDR-plus ELISA represents a reliable and specific serological tool for detecting antibodies against *L. infantum* in endemic regions, particularly in high seropositive dogs, while also reliably yielding negative results in seronegative animals. However, its diagnostic performance decreases in healthy seropositive dogs and those with low antibody levels, as these are the most challenging clinical–serological profiles to detect. The combination of the rKDDR-plus ELISA with molecular or complementary serological techniques could further enhance diagnostic accuracy and broaden its applicability in both clinical and epidemiological settings.

## Figures and Tables

**Figure 1:**
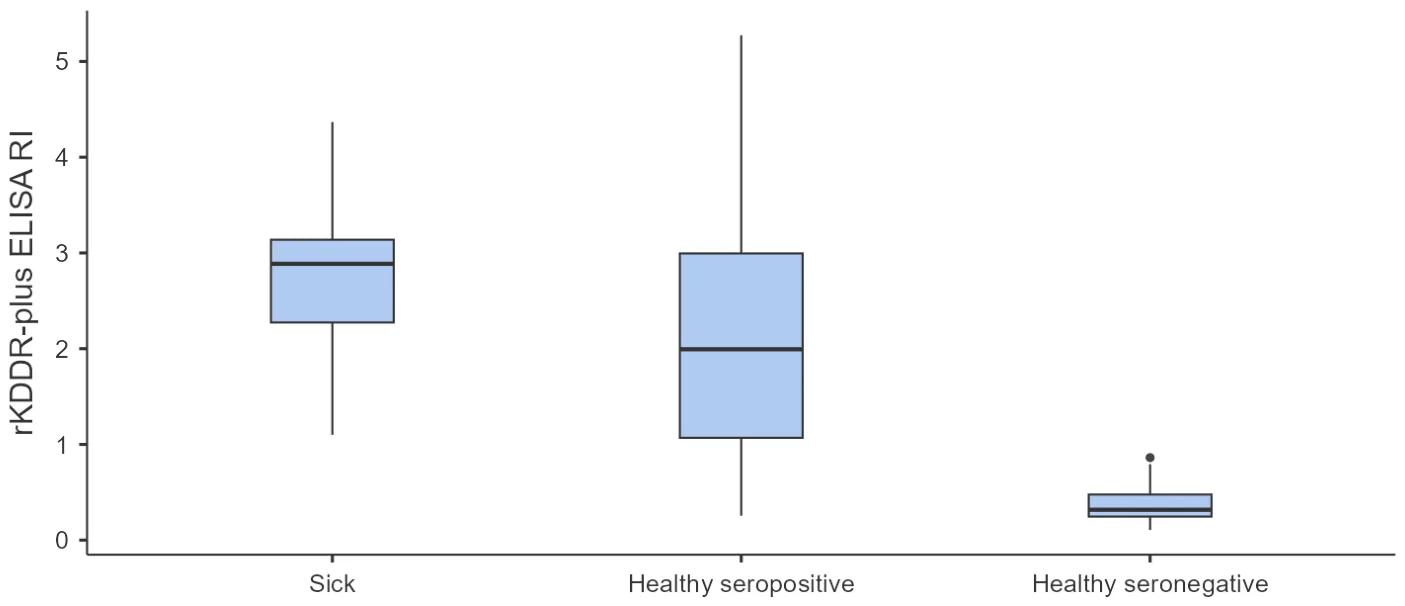
Distribution of rKDDR-plus ELISA RI values according to the different clinical and serological profiles of dogs. Abbreviation: RI, reactivity index. The boxes represent the interquartile range (IQR), extending from the 25th percentile (Q1) to the 75th percentile (Q3), and the horizontal line within each box represents the median. The whiskers extend to the lowest and highest values within 1.5 times the IQR from Q1 and Q3, respectively. Points beyond the whiskers represent outliers.

**Figure 2:**
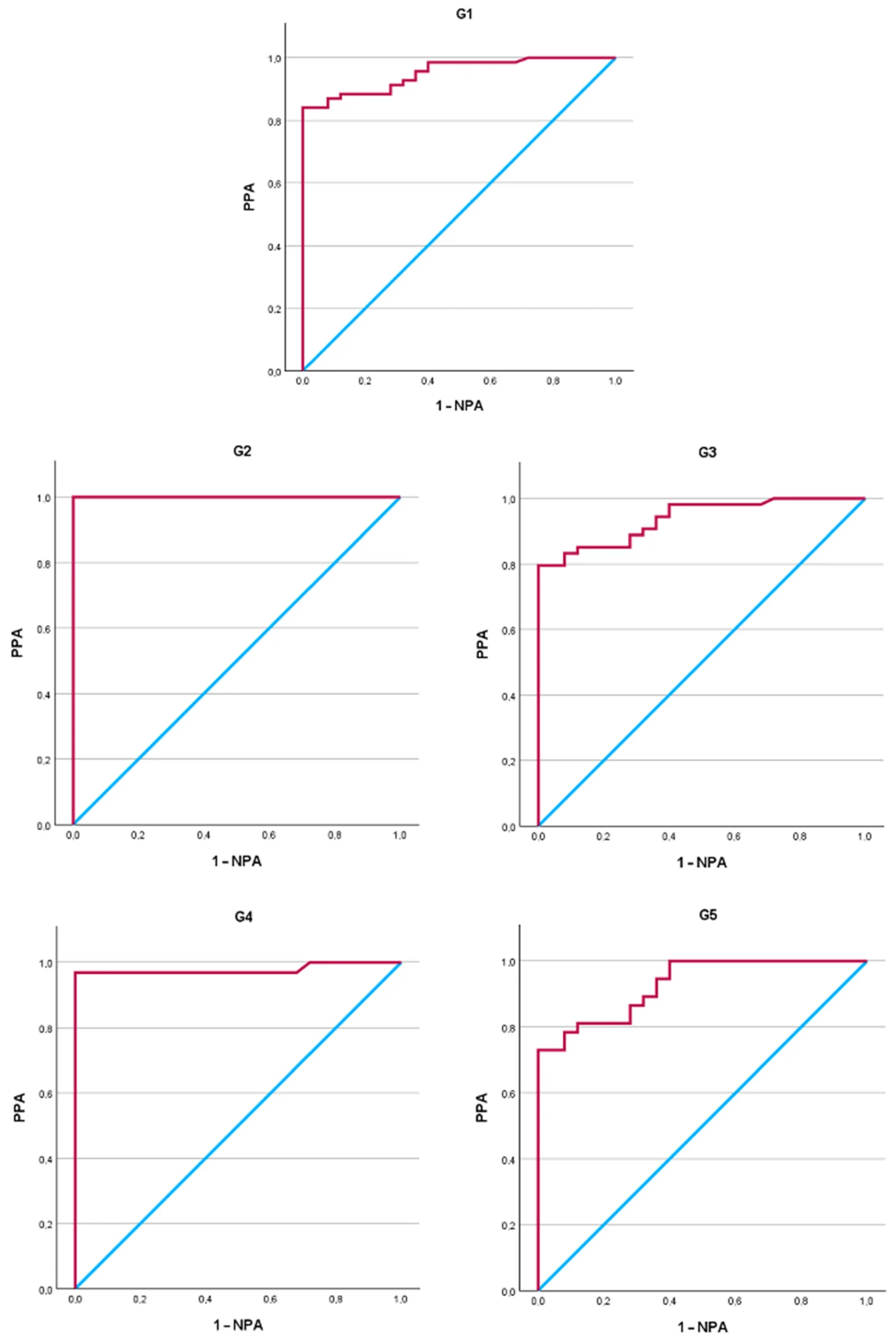
Receiver operating characteristic curves (dark red line) and area under the curve (AUC) of rKDDR-plus ELISA for all groups analyzed. G1: all dogs (*n* = 94), AUC 0.951 (95% CI: 0.912–0.990); G2: healthy seronegative + sick (*n* = 40), AUC 1.000 (95% CI: 1.000–1.000); G3: healthy seronegative + healthy seropositive (*n* = 79), AUC 0.937 (95% CI: 0.889–0.986); G4: healthy seronegative + high seropositive (*n* = 57), AUC 0.978 (95% CI: 0.935–1.000 *); G5: healthy seronegative + low seropositive (*n* = 62), AUC 0.928 (95% CI: 0.868–0.987). Blue line: random classifier (chance diagonal). Abbreviations: PPA, positive percent agreement; NPA, negative percent agreement. * The asymptotic upper confidence limit generated by SPSS was 1.021; for reporting purposes, it was constrained to the theoretical upper bound of the AUC (1.000).

**Table 1:** Demographic characteristics of the dogs studied in different states of *L. infantum* infection and in-house ELISA results.

Qualitative Variables		Number of Dogs (%)			
		Healthy Seronegative (*n* = 25)	Healthy Seropositive (*n* = 54)	Sick (*n* = 15)	Total (*n* = 94)
Sex ^a^	Male	9 (50.0)	31 (59.6)	8 (53.3)	48 (56.5)
	Female	9 (50.0)	21 (40.4)	7 (46.7)	37 (43.5)
Neutering ^b^	Yes	4 (36.4)	17 (37.8)	4 (30.8)	25 (36.2)
	No	7 (63.6)	28 (62.2)	9 (69.2)	44 (63.8)
Age	Young	2 (11.8)	3 (6.0)	2 (13.3)	7 (8.5)
	Adult	9 (52.9)	33 (66.0)	12 (80.0)	54 (65.9)
	Geriatric	6 (35.3)	14 (28.0)	1 (6.7)	21 (25.6)
Breed ^c^	Purebred	13 (76.5)	28 (57.1)	7 (46.7)	48 (59.3)
	Crossbreed	4 (23.5)	21 (42.9)	8 (53.3)	33 (40.7)
	Most common breeds	Hound 4 (23.5)	Hound 5 (10.2) Pitbull 3 (6.1)	Spanish Bulldog, American Bull Terrier, American Staffordshire terrier, Belgian Malinois shepherd, mastiff, hound, rottweiler, 1 each (6.7)	Hound 10 (12.3)
		Mastiff 2 (11.8)			Mastiff 3 (3.7)
		German Sheperd 2 (11.8)			Pitbull 3 (3.7)
Diagnostic ELISA interpretation	High positive	NA	24 (44.4)	8 (53.3)	32 (46.4) *
	Low positive	NA	30 (55.6)	7 (46.7)	37 (53.6) *
Quantitative variables		Median (IQR)			
Age (years) ^d^		6 (5.0)	5 (4.0)	4 (3.8)	5 (4.8)
Weight (kg) ^e^		27 (15.0)	24 (12.2)	22.8 (19.6)	25 (11.9)
Diagnostic ELISA (EU)		16.6 (10.6)	137.9 (145.3)	167.14 (144.2)	97.89 (176.4)
Endpoint ELISA (EU)		NA	176.13 (313.6)	202.69 (327.2)	125.24 (229.3)

^a^ Sex was not recorded in 9 dogs. ^b^ Reproduction status was not recorded in 25 dogs. ^c^ The breed was not recorded in 13 dogs. ^d^ Age was not recorded in 12 dogs. ^e^ Weight was not recorded in 17 dogs. * EU interpretation from the total of positive dogs. Abbreviations: EU, ELISA units; IQR, interquartile range; NA, not applicable.

**Table 2:** Median of ELISA Units (EU) and seroreactivity rate according to endpoint and diagnostic *L. infantum* ELISA and rKDDR-plus serological ELISA.

Parameter Number of Dogs (%)		Endpoint ELISA Median (IQR)	Diagnostic ELISA Number of Positive Dogs (%)	rKDDR-Plus ELISA Number of Positive Dogs (%)
Sex ^a^	Male 48 (56.5)	154 (354)	39 (81.3)	31 (64.6)
	Female 37 (43.5)	123 (207)	28 (75.7)	24 (64.9)
Neutering ^b^	Yes 25 (36.2)	145 (314)	21 (84.0)	18 (72.0)
	No 44 (63.8)	155 (171)	37 (84.1)	29 (65.9)
Age ^c^	Young 7 (8.5)	111.4 (212)	5 (71.4)	5 (71.4)
	Adult 54 (65.9)	155.2 (309)	45 (83.3)	38 (70.4)
	Geriatric 21 (25.6)	125.2 (160)	15 (71.4)	10 (47.6)
Breed ^d^	Purebred 48 (59.3)	116 (299)	35 (72.9)	26 (54.2)
	Crossbreed 33 (40.7)	146.9 (153)	29 (87.9)	26 (78.8)
Clinical condition ^e^	Healthy seronegative 25 (26.6)	16.6 (10.6) ^f,g^		
	Healthy seropositive 54 (57.4)	176.1 (313.6) ^f^		
	Sick 15 (16.0)	202.7 (327.2) ^g^		

^a^ Sex was not recorded in 9 dogs. ^b^ Reproduction status was not recorded in 25 dogs. ^c^ Age was not recorded in 12 dogs. ^d^ Breed was not recorded in 13 dogs. ^e^ Kruskal–Wallis H-test: χ^2^ = 54.26, *p* < 0.001. Dwass–Steel–Critchlow–Fligner pairwise comparison ^f^ and ^g^ *p* < 0.001. Abbreviations: EU, ELISA units; IQR; interquartile range.

**Table 3:** Diagnostic performance of the rKDDR-plus serological ELISA test.

Group	PPA (%) [CI 95%]	NPA (%) [CI 95%]	Youden Index	Kappa	Agreement
G1	82.61 [71.59–90.68]	100 [86.28–100]	0.83	0.716	Substantial
G2	100 [78.2–100]	100 [86.28–100]	1	1	Almost perfect
G3	77.78 [64.4–87.96]	100 [86.28–100]	0.78	0.689	Substantial
G4	96.88 [83.78–99.92]	100 [86.28–100]	0.97	0.965	Almost perfect
G5	70.27 [53.02–84.13]	100 [86.28–100]	0.70	0.656	Substantial

G1: all dogs (*n* = 94); G2: healthy seronegative + sick (*n* = 40); G3: healthy seronegative + healthy seropositive (*n* = 79); G4: healthy seronegative + high seropositive (*n* = 57); G5: healthy seronegative + low seropositive (*n* = 62). Abbreviations: CI, confidence interval; NPA, negative percent agreement; PPA, positive percent agreement.

**Table 4:** Positive predictive value, negative predictive value and accuracy of the rKDDR-plus serological ELISA test considering different seroprevalence scenarios.

	Group	PPV (%) [CI 95%]	NPV (%) [CI 95%]	AC (%) [CI 95%]
5% prevalence				
	G1	100 [93.73–100]	99.09 [98.49–99.46]	99.13 [94.54–99.99]
	G2	100 [78.2–100]	100 [86.28–100]	100 [91.19–100]
	G3	100 [91.59–100]	98.84 [98.11–99.29]	98.89 [93.41–99.98]
	G4	100 [88.78–100]	99.84 [98.88–99.98]	99.84 [93.42–100]
	G5	100 [86.77–100]	98.46 [97.50–99.06]	98.51 [91.55–99.97]
10% prevalence				
	G1	100 [93.73–100]	98.10 [96.87–98.86]	98.26 [93.12–99.85]
	G2	100 [78.2–100]	100 [86.28–100]	100 [91.19–100]
	G3	100 [91.59–100]	97.59 [96.09–98.52]	97.78 [91.62–99.78]
	G4	100 [88.78–100]	99.65 [97.67–99.95]	99.69 [93.12–100]
	G5	100 [86.77–100]	96.80 [94.86–98.03]	97.03 [89.21–99.68]
20% prevalence				
	G1	100 [93.73–100]	95.83 [93.22–97.47]	96.52 [90.55–99.21]
	G2	100 [78.2–100]	100 [86.28–100]	100 [91.19–100]
	G3	100 [91.59–100]	97.56 [91.62–96.74]	95.56 [88.39–98.91]
	G4	100 [88.78–100]	99.23 [94.9–99.89]	99.38 [92.54–100]
	G5	100 [86.77–100]	93.08 [89.13–95.67]	94.05 [84.98–98.47]
30% prevalence				
	G1	100 [93.73–100]	93.06 [88.92–95.73]	94.78 [88.16–98.31]
	G2	100 [78.2–100]	100 [86.28–100]	100 [91.19–100]
	G3	100 [91.59–100]	91.30 [86.44–94.53]	93.33 [85.40–97.72]
	G4	100 [88.78–100]	98.68 [91.56–99.81]	99.09 [91.99–100]
	G5	100 [86.77–100]	88.70 [82.71–92.80]	91.08 [81.08–96.83]
50% prevalence				
	G1	100 [93.73–100]	85.19 [77.47–90.58]	91.30 [83.69–96.13]
	G2	100 [78.2–100]	100 [86.28–100]	100 [91.19–100]
	G3	100 [91.59–100]	81.82 [73.21–88.11]	88.89 [79.81–94.85]
	G4	100 [88.78–100]	96.97 [82.30–99.55]	98.44 [90.92–99.97]
	G5	100 [86.77–100]	77.08 [67.21–84.66]	85.14 [73.81–92.89]

G1: all dogs (*n* = 94); G2: healthy seronegative + sick (*n* = 40); G3: healthy seronegative + healthy seropositive (*n* = 79); G4: healthy seronegative + high seropositive (*n* = 57); G5: healthy seronegative + low seropositive (*n* = 62). Abbreviations: AC, accuracy; CI, confidence interval; NPV, negative predictive value; PPV, predictive positive value.

**Table 5:** Receiver operating characteristic (ROC) analysis of the rKDDR-plus ELISA using continuous relative index (RI) values in the different groups of dogs studied.

Group	AUC [CI 95%]	SE	Optimal RI Cutoff	PPA at Optimal Cutoff	NPA at Optimal Cutoff	Maximum Youden Index
G1	0.951 [0.912–0.990]	0.020	0.9290	84.1%	100%	0.841
G2	1.000 [1.000–1.000]	0.000	0.9810	100%	100%	1.000
G3	0.937 [0.889–0.986]	0.025	0.9290	79.6%	100%	0.796
G4	0.978 [0.935–1.000] *	0.022	0.9325	96.9%	100%	0.969
G5	0.928 [0.868–0.987]	0.030	0.9290	73.0%	100%	0.730

* As AUC values are theoretically bounded between 0 and 1, asymptotic confidence limits falling outside this range were constrained to the corresponding theoretical boundary for reporting. G1: all dogs (*n* = 94); G2: healthy seronegative + sick (*n* = 40); G3: healthy seronegative + healthy seropositive (*n* = 79); G4: healthy seronegative + high seropositive (*n* = 57); G5: healthy seronegative + low seropositive (*n* = 62). Abbreviations: AUC, area under the curve; CI, confidence interval; NPA, negative percent agreement; PPA, positive percent agreement; RI, reactivity index; SE, standard error.

## Data Availability

The original contributions presented in this study are included in the article/Supplementary Materials. Further inquiries can be directed to the corresponding author.

## Supplementary Materials

The following supporting information can be downloaded at https://www.mdpi.com/article/10.3390/ani16193097/s1. All data supporting the main conclusions are available in the manuscript and in the Supplementary Material (Dataset S1: signalment, clinical data and ELISA results).

## Abbreviations

The following abbreviations are used in this manuscript:

AC	Accuracy
AUC	Area under the curve
CI	Confidence interval
ELISA	Enzyme-linked immunosorbent assay
EU	ELISA units
ICT	Immunochromatography
IFAT	Indirect fluorescent antibody test
IQR	Interquartile range
NA	Not applicable
NPA	Negative percent agreement
NPV	Negative predictive value
OD	Optical density
OPD	o-Phenylenediamine dihydrochloride
PBS	Phosphate buffered saline
PPA	Positive percent agreement
PPV	Predictive positive value
RI	Reactivity Index
ROC	Receiver operating characteristic
Th	T helper
